# Pastoral Practices to Reverse Shrub Encroachment of Sub-Alpine Grasslands: Dung Beetles (Coleoptera, Scarabaeoidea) Respond More Quickly Than Vegetation

**DOI:** 10.1371/journal.pone.0083344

**Published:** 2013-12-16

**Authors:** Claudia Tocco, Massimiliano Probo, Michele Lonati, Giampiero Lombardi, Matteo Negro, Beatrice Nervo, Antonio Rolando, Claudia Palestrini

**Affiliations:** 1 Department of Life Sciences and System Biology, University of Torino, Torino, Italy; 2 Department of Agricultural, Forest and Food Sciences, University of Torino, Grugliasco (Torino), Italy; National University of Mongolia, Mongolia

## Abstract

In recent decades, pastoral abandonment has produced profound ecological changes in the Alps. In particular, the reduction in grazing has led to extensive shrub encroachment of semi-natural grasslands, which may represent a threat to open habitat biodiversity. To reverse shrub encroachment, we assessed short-term effects of two different pastoral practices on vegetation and dung beetles (Coleoptera, Scarabaeoidea). Strategic placement of mineral mix supplements (MMS) and arrangement of temporary night camp areas (TNCA) for cattle were carried out during summer 2011 in the Val Troncea Natural Park, north-western Italian Alps. In 2012, one year after treatment, a reduction in shrub cover and an increase in bare ground cover around MMS sites was detected. A more intense effect was detected within TNCA through increases in forage pastoral value, and in the cover and height of the herbaceous layer. Immediately after treatment, changes in dung beetle diversity (total abundance, species richness, Shannon diversity, taxonomic and functional diversity) showed a limited disturbance effect caused by high cattle density. In contrast, dung beetle diversity significantly increased one year later both at MMS and TNCA sites, with a stronger effect within TNCA. Multivariate Regression Trees and associated Indicator Value analyses showed that some ecologically relevant dung beetle species preferred areas deprived of shrub vegetation. Our main conclusions are: i) TNCA are more effective than MMS in terms of changes to vegetation and dung beetles, ii) dung beetles respond more quickly than vegetation to pastoral practices, and iii) the main driver of the rapid response by dung beetles is the removal of shrubs. The resulting increase in dung beetle abundance and diversity, which are largely responsible for grassland ecosystem functioning, may have a positive effect on meso-eutrophic grassland restoration. Shrub encroachment in the Alps may therefore be reversed, and restoration of grassland enhanced, by using appropriate pastoral practices*.*

## Introduction

Agricultural abandonment of mountain areas has been an increasing post-war trend in Western Europe [1]. The reduction in grazing at many locations has resulted in natural successional phenomena with changes in vegetative structure and composition [2]. In the sub-alpine (below the treeline) and alpine (above the treeline) belts, the encroachment of semi-natural grasslands by trees and dwarf shrubs (predominantly *Rhododendron ferrugineum* L., *Juniperus nana* Willd., *Vaccinium myrtillus* L., and *Vaccinium gaultherioides* Bigelow) has reduced the extent of such meso-eutrophic open habitats, causing a decline in forage yield and quality, and a reduction in landscape heterogeneity [1,3]. The loss of semi-natural grasslands is also threatening alpine biodiversity, as the preservation of many plant and animal species is strongly dependent on the maintenance of open habitats [4–7]. In many European countries, management to reverse shrub encroachment and restore grassland has been mainly carried out by manual or mechanical shrub clearing, mowing, prescribed burning, and grazing [8–10]. Livestock management plays an important role in the restoration process, as the combined effect of animal trampling, grazing, seed transport, and nutrient redistribution through dung deposition affects the characteristics of habitats, such as the structure of vegetation, the botanical composition, and soil features [9]. Moreover, concentration of nutrients due to the deposition of urine and faeces by livestock favors the development of meso-eutrophic herbaceous vegetation types, characterized by a high frequency of nutrient-demanding species [11]. However, not all grazing regimes are equally effective. Free-ranging herbivores, for instance, are not able to prevent encroachment by shrubs and trees [12]. Therefore, to use grazing management as a tool for alpine grassland restoration requires knowledge of the most effective strategies to be employed. 

Many of the effects produced by livestock are mediated by dung beetles (Coleoptera, Scarabaeoidea). Their feeding and nesting behaviour can cause a removal and reduction in the amount of dung in the short-term [13] and contribute to increased seed dispersal, preventing the loss of primary and secondary nutrients and enhancing soil fertility, porosity, aeration, and water infiltration [14,15]. The displacement and mixing of sediment particles by tunneller dung beetles can increase soil aeration, accelerating bacterial growth responsible for N mineralization [16]. Moreover, the activity of burying dung might reduce the volatilization of NH_3_, and enhance N utilization by plants, especially in the first 10 cm of soil [14]. 

This study focuses upon the potential to reverse shrub-encroachment and to restore open habitats by two different pastoral practices: the strategic placement of mineral mix supplements (MMS) and the arrangement of temporary night camp areas (TNCA) for cattle. The strategic placement of MMS is a method to entice cattle into traditionally underused areas [17], whereas TNCA is a modification of a traditional alpine practice in which livestock were usually confined within herbaceous camp areas controlled by farmers at night, in order to prevent attacks from wolves and cattle rustling. In contrast with this traditional practice, our night camp areas were temporary and placed over shrub-encroached vegetation. Areas around MMS and within TNCA were expected to undergo the effects of a very high grazing pressure, i.e. trampling and important deposition of livestock excreta (urine and dung), and consequently large inputs of nutrients, which may produce a shift in plant [11] and dung beetle [18] communities. Given the relevance of dung beetles to grassland ecology, to evaluate the restoration potential of these practices we considered both the effects on vegetation and on dung beetle assemblages. We also assumed dung beetles are a key group to monitor the results of pastoral restoration practices in the Alps because habitat modifications have immediate and severe consequences for their assemblages [18,19]. 

The general aim of this work was to assess the effects of MMS and TNCA for the control of shrub encroachment and the restoration of open habitats on the short-term. Immediate effects on vegetation are largely unknown because research usually focuses on medium and long-term effects given the slow response of vegetation at high altitudes [20]. Nevertheless, the implementation of many restoration practices on moderate to steep slopes (as the ones generally encroached by shrubs) might result in an increased risk of erosion and solifluction, which only a prompt recovery by herbaceous species could contrast. Thus an assessment of open habitat restoration on the short-term could be very important, especially with the view of an application to a larger scale. More specific aims were: i) to compare the effects of the two practices on vegetation and dung beetles in order to identify the practice which is more efficient to reverse shrub encroachment; ii) to contrast, in the context of each practice, the effects on vegetation and dung beetles, in order to identify the component which responds and recovers faster, and iii) to identify which vegetation variable, if any, influences dung beetle communities.

## Materials and Methods

The Val Troncea Natural Park gave permission to conduct this research. The approval of the Institutional Animal Care and Use Committee (IACUC) was not required in this study as cattle were only subjected to conventional pastoral practices (use of supplements and fencing). 

### Study area

**Figure 1:**
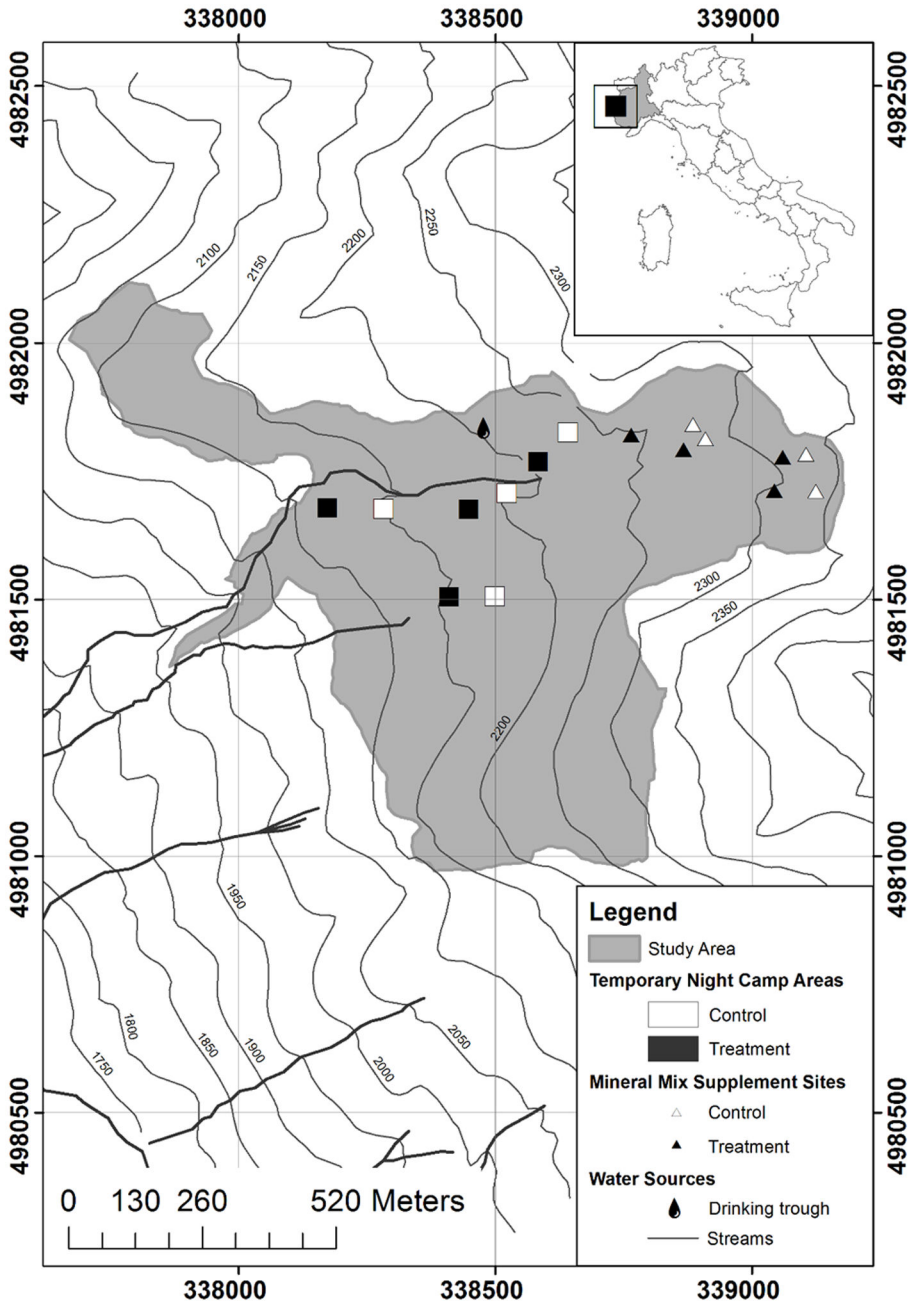
Study area. Location of the study area in Val Troncea, Western Alps (inset), Piedmont, Italy (UTM zone 32 north, WGS84 datum).

The study was conducted in Val Troncea Natural Park (Figure 1), Piedmont, south-western Alps (latitude 44° 57’ N, longitude 6° 57’ E). Throughout the last decades, the Val Troncea Natural Park has experienced changes in shrubland extent and is therefore representative of habitat subject to shrub encroachment and loss of grasslands due to pastoral abandonment. Annual average air temperature was 0.8 °C (January: -8 °C; July: 9.5°C) and annual average precipitation was 956 mm. The study area consisted of a large enclosure (about 75 hectares), which was the most shrub-encroached one out of the 18 enclosures managed under a rotational grazing system during summer by one of the two cattle farms still operating within the Park (Figure 1). Within the enclosure, where elevations ranged from 1960 to 2360 m a.s.l., the most extended grassland and shrubland communities were dominated by *Festuca curvula* Gaudin, *J.nana*, *Geum montanum* L. and *Nardus stricta* L., *R. ferrugineum, Calamagrostis villosa* (Chaix) J. F. Gmel and *Festuca flavescens* Bellardi, and *Festuca* gr. *rubra* and *Agrostis tenuis* Sibth. The study area was grazed for 22 days (from 28 June to 18 July 2011) by 160 beef cows, corresponding to 135 livestock units (LU), predominantly of the Piedmontese breed, but also with some Valdostana Red Pied and Barà-Pustertaler breeds. The group included heifers and non-lactating cows, varying in age from 1 to 15 years. 

### Placement of MMS and TNCA

Four MMS sites and four TNCA sites were placed within the study area in the sub-alpine belt (Figure 1). MMS sites were positioned within large patches of shrub-encroached grasslands with roughly the same slope (30 %, on average). Within each site, cows were offered phosphate mineral mix supplements *ad libitum* for the whole grazing period (i.e. from 28 June to 18 July 2011). MMS were supplied in 5-kg blocks, which were placed 5 m apart in pairs. 

TNCA sites were positioned within large patches of shrub-encroached grasslands with roughly the same slope (28% on average). TNCA were arranged from 30 June to 15 July 2011 and cattle were confined for two consecutive nights within each area, which was delimited with electric fences. The extent of TNCA was on average 1107 m^2^. An area of about 7 m^2^ per night was available to each cow, resulting in a stocking density of 0.12 LU m^-2^.

Each pair of MMS blocks and each TNCA was considered as a treatment site and was paired with a control site placed at a maximum distance of 130 m. Control sites of MMS and TNCA had approximately the same slope, area, soil cover, vegetation features, and distances from water sources with respect to the relative treatment sites.

### Vegetation surveys

Botanical composition was determined using the vertical point-quadrat method [21] along cross-shaped transects. One transect was placed at each control and treatment site, with the centre of the cross positioned in the midpoint between the two MMS blocks and in the barycentre of the TNCA. Transects were 12.5 m long across each of the four sides of the cross and surveys were carried out in late June 2011 (i.e. pre-treatment) and 2012 (i.e. one year after treatment). In each transect, at every 50-cm interval, plant species touching a steel needle were identified and recorded (i.e. 100 points of vegetation measurement per transect). Since rare species are often missed by this method, a complete list of all other plant species included within a 1-m buffer around the transect line was also recorded [21]. Within the same buffer, the percentage of shrub, herbaceous, and bare ground cover was visually estimated and 20 measurements of the height of the shrub and the herbaceous layers were randomly carried out with the ‘sward stick method’ [22]. In late July 2011 (i.e. immediately after treatments), the extent of areas modified by cattle around MMS locations was visually estimated, following the sharp fine scale fragmentation of the original dense shrub cover. 

### Dung beetle sampling

Dung beetles were sampled using a total of 80 pitfall traps: four at each MMS site, six at each TNCA, and the same number for their paired control sites. Pitfall traps were of the hang bait type, baited with 250 g of fresh cow dung. At MMS and paired control sites, the four pitfall traps were placed at four corners of a 20 x 20 m plot with the two MMS blocks at the centre. At TNCA and paired control sites the six pitfall traps were randomly placed at a distance of about 20 m from one another. All pitfall traps were located within a 5 m buffer from vegetation transects. In 2011, traps were placed after the end of treatments and the sampling activity lasted from late July to late September. Sampling was also repeated one year after treatments, from late July to late September 2012. All traps were emptied and re-baited every 2 weeks, giving rise to 5 different sampling periods every year.

Beetles were identified to the species level.

### Data analysis

#### Vegetation variables

For each plant species recorded in the vegetation transects the frequency of occurrence (f_i_ = number of occurrences/100 points), which is an estimate of species canopy cover [23], was calculated. For each transect, average heights of the shrub and the herbaceous layers were calculated. Vegetation diversity was expressed according to three indices: species richness, Shannon diversity index, and Pielou’s equitability index [24]. Shannon diversity was measured as the exponential of the Shannon-Weaver index (Shannon entropy) [25]. Each plant species was classified according to the Landolt nutrient value (index *N*, [26]), as oligotrophic (*N* = 1, 2), mesotrophic (*N* = 3), or eutrophic (*N* = 4, 5), and the frequencies of oligotrophic, mesotrophic, and eutrophic species were calculated. The average vegetation *N* index was calculated for each transect to evaluate the overall effect of fertilization produced directly or indirectly (i.e. *via* dung beetles). In each transect, forage pastoral value, a synthetic value which summarizes forage yield and nutritive value, was calculated on the basis of the species’ frequencies and the Index of Specific Quality [3]. 

#### Dung beetle variables

Dung beetle diversity was expressed by using the following community parameters: total abundance, species richness, and Shannon diversity index [25]. Three taxonomic diversity indices quantifying differences between the species within a sample were also calculated: taxonomic diversity, taxonomic distinctness, and average taxonomic distinctness. These three indices are independent of sample effort, unbiased, related to functional diversity and sensitive to environmental impacts, so they may represent useful parameters in terms of bio-diagnostic purposes [27,28]. 

We classified all species according to four functional traits using an ecomorphological approach in order to calculate the functional diversity index [29]. Functional traits used were nesting behaviour (according to [13,30,31]), weight, and two protoracical leg allometries (Table S1 in Appendix S1). 

Further details about the vegetation and dung beetle variables are shown in Appendix S1.

#### Statistical analyses

For dung beetles, a completeness analysis of sampling in each treatment and control plot was conducted by computing abundance-based estimates using two variants of extrapolated richness: Chao (unbiased variant) and abundance-based richness estimator (ACE) [32]. The completeness inventory for each plot was measured as the percentage of the total number of species predicted by the estimators that were actually recorded. 

Generalized Linear Mixed Models (GLMMs, [33]) were used to test for differences between treatment and control plots in vegetation variables and in dung beetle variables. In all dung beetle analyses performed, the trap was used as the sampling unit. In all GLMMs, treatment was considered as a fixed factor. The random effects considered were study area (where each study area contains both treatment and control site, thus maintaining the paired structure) in vegetation analyses and area and sampling period in dung beetle analyses. Moreover, for vegetation, in order to eliminate variations related to pre-treatment vegetation status between treatment and control sites, the ratio between years (2012 over 2011) was calculated for variables with residuals following a gamma distribution, and by specifying 2011 as an offset for the other variables [23,33]. A Poisson distribution was specified for count variables which were not overdispersed (overdispersion in the data was tested by the qcc R package, [34]), a Negative Binomial distribution was specified for count variables with overdispersion, and a gamma distribution was specified for continuous data, as normality was not met (normality was tested with Kolmogorov-Smirnoff test, [33]). Significance tests were performed using the Wald statistic.

The variation in dung beetle assemblage structure among sampling periods and vegetation variables was evaluated using Multivariate Regression Trees (MRTs) [35]. MRT was performed on 2012 data in order to assess the relationships between vegetation and dung beetle species one year post-treatment (i.e. short-term effects of treatments). For this reason, only vegetation variables which were significantly different between treatment and control sites were used (Table 1). We combined MRT (using the trap as the sampling unit) with Indicator Value calculations [36] for the species falling into the MRT branches. 

**Table 1:** Effects of pastoral practices on vegetation.

			**MMS**			**TNCA**		
	**Vegetation variables**	**Distribution**	**Est.**	**Wald**	***p***	**Est.**	**Wald**	***p***
Vegetation structure variables	**SC**	Gamma	**-0.4**	**-3.8**	**<0.001**	**-0.74**	**-4.04**	**<0.001**
	**HC**	Gamma	0.01	0.07	N.S.	**0.21**	**2.06**	**<0.05**
	**BGC**	Gamma	**1.55**	**10.77**	**<0.001**	**1.15**	**7.07**	**<0.001**
	**AHHL**	Gamma	0.12	0.91	N.S.	0.26	1.96	**<0.1**
	**AHSL**	Gamma	-0.03	-0.41	N.S.	0.08	1.52	N.S.
Biodiversity indices	**S_V_**	Poisson	-0.01	0.08	N.S.	0.07	1.11	N.S.
	**H_V_**	Gamma	-0.09	-0.53	N.S.	0.27	2.25	**<0.05**
	**Jv**	Gamma	-0.01	-0.12	N.S.	0.08	1.48	N.S.
Species frequencies	**OS**	Poisson	-0.22	-3.59	N.S.	-0.08	-1.41	N.S.
	**MS**	Neg Binomial	-0.51	-1.3	N.S.	0.05	0.15	N.S.
	**ES**	Neg Binomial	0.77	1.14	N.S.	-0.18	-0.7	N.S.
	**Naverage**	Gamma	0.03	1.42	N.S.	0.01	0.32	N.S.
	**PV**	Gamma	0.09	0.37	N.S.	**0.34**	**2.66**	**<0.01**

Treatment factor estimates and statistical significance (GLMM) for vegetation variables between treatment (MMS and TNCA) and control sites: vegetation structure (SC, HC, and BCG represent the percentage of shrub, herbaceous, and bare ground cover, respectively; AHSL and AHHL the average heights of the shrub and herbaceous layers), biodiversity indices (S_V_, species richness; H_V_, Shannon diversity index; J_V_, equitability index), species frequencies (OS, oligotrophic species; MS; mesotrophic; ES, eutrophic species), average vegetation *N* index (N_average_), and forage pastoral value (PV). Significant comparisons are in bold type. In this parameter estimation analysis, the control sites were used as the reference category.

All analyses were carried out using R 2.15.1 with labdsv, mvpart, glmmADMB, and Vegan packages [37].

## Results

### Effects on vegetation

A total of 97 species was detected in botanical surveys. Dominant species were *J. nana*, *R. ferrugineum*, *V. myrtillus*, *Poa chaixii* (Vill.), and *Avenella flexuosa* L. 

Areas modified by cattle around MMS sites had an elliptic shape, with the main axis placed along level curves, and with an average extent of 69.1 m^2^. One year after the use of MMS by cattle, percentage of shrub cover decreased and the percentage of bare ground cover increased with respect to the control sites (Table 1). Indeed, within MMS treatment sites the average percentage of shrub cover decreased from 74 to 49 % and the average percentage of bare ground cover increased from 7 to 33 %. One year after the arrangement of TNCA, the percentage of shrub cover was lower, while the percentage of herbaceous cover, the percentage of bare ground cover, and the average height of the herbaceous layer were higher within the TNCA than at the control sites. Shannon diversity index and forage pastoral value were also higher at the TNCA (Table 1). Within TNCA treatment sites, the percentage of shrub cover decreased from 57 to 29 %, the percentage of herbaceous cover increased from 33 to 40 %, the percentage of bare ground cover increased from 10 to 31 %, the average height of the herbaceous layer increased from 9.5 to 13.2 cm, Shannon diversity index increased from 22.8 to 31.6, and forage pastoral value increased from 8.2 to 11.6, on average.

### Effects on dung beetles

A total of 22 species (31 422 individuals) of three families (Aphodiidae, Scarabeidae, and Geotrupidae) was collected (Table 2). Completeness analysis values ranged from 72 to 99 % irrespective of the estimator employed (Table 2). As most of the expected species were collected, it was assumed that the sampling effort was sufficient. The assemblage, as a whole, was dominated by Aphodiidae (93 % of the sampled individuals), followed by Scarabaeidae and Geotrupidae (6 and 1 %, respectively). Dominant species were *Planolinus fasciatus* Olivier, *Euheptaulacus carinatus* Germar, *Colobopterus erraticus* L., and *Amidorus obscurus* Fabricius.

**Table 2:** Sampled dung beetles.

	**MMS**				**TNCA**			
**Sampled dung beetles**	2011	2011	2012	2012	2011	2011	2012	2012
	Treat.	Cont.	Treat.	Cont.	Treat.	Cont.	Treat.	Cont.
**Geotrupidae Latreille, 1802**								
*Anoplotrupes stercorosus* (Scriba)	0	0	2	0	0	0	14	0
*Geotrupes stercorarius* (Linnaeus)	16	4	17	3	27	36	116	7
*Trypocopris alpinus* (Sturm & Hagenbach)	1	11	37	4	0	0	13	0
**Aphodiidae Leach,1815**								
*Acrossus depressus*(Kugelann)	0	0	2	4	2	2	53	13
*Acrossus rufipes*(Linnaeus)	24	20	22	13	74	151	187	112
*Agolinus satyrus* (Reitter)	31	21	18	19	138	261	635	116
*Aphodius fimetarius* (Linnaeus)	2	6	0	0	4	8	46	0
*Amidorus immaturus*(Mulsant)	52	70	29	18	27	19	46	11
*Amidorus obscurus* (Fabricius)	366	453	214	115	172	159	248	57
*Bodilopsis rufa* (Moll)	10	5	99	46	64	53	405	155
*Colobopterus erraticus*(Linnaeus)	225	238	282	217	189	366	724	198
*Coprimorphus scrutator* (Herbst)	0	0	0	0	0	0	12	0
*Euheptaulacus carinatus* (Germar)	1643	1475	869	511	656	1264	1602	589
*Esymus pusillus* (Herbst)	4	2	14	5	29	42	130	56
*Otophorus haemorrhoidalis* (Linnaeus)	112	89	139	52	277	349	255	108
*Oromus alpinus* (Scopoli)	4	2	14	3	3	0	346	45
*Parammoecius corvinus* (Erichson)	1	1	3	0	23	17	127	11
*Planolinus fasciatus* (Olivier)	87	49	71	58	2992	3656	2587	1345
*Rhodaphodius foetens* (Fabricius)	0	1	0	0	2	4	19	0
*Teuchestes fossor* (Linnaeus)	0	1	2	0	3	8	43	2
**Scarabaeidae Latreille,1802**								
*Onthophagus baraudi* (Nicolas)	16	23	132	103	8	16	113	76
*Onthophagus fracticornis* (Preyssler)	129	224	291	192	75	126	390	70

**Total number (N_DB_)**	2723	2695	2257	1363	4765	6537	8111	2971
**Species richness (S_DB_)**	16	18	18	15	18	17	21	16
**Completeness chao1 (%)**	89.58	78.23	98.44	91.53	96.43	95.79	91.67	99.19
**Completeness ACE (%)**	86.38	71.95	96.12	84.64	86.12	90.07	88.73	96.37

Numbers of dung beetle individuals, species richness, average species estimates (chao1 and ACE), and sample completeness for both treatments (placement of mineral mix supplements, MMS and arrangement of temporary night camp areas, TNCA) and control sites in both sampling years. Treat: Treatment sites; Cont: Control sites.

For MMS, only taxonomic diversity and taxonomic distinctness were higher at control sites in 2011, while in 2012 the pattern was reversed: total abundance, species richness, taxonomic diversity, and functional diversity index were significantly higher at treatment than at control sites, while Shannon diversity index was significantly lower (Table 3). For TNCA, total abundance, taxonomic distinctness, average taxonomic distinctness, and functional diversity index were lower at treatment than at control sites in 2011, while in 2012 the pattern was totally reversed: total abundance, species richness, Shannon diversity index, taxonomic diversity, average taxonomic distinctness, and functional diversity index were significantly higher at treatment than at control sites (Table 3). It is worth mentioning that in 2012, TNCA areas were intensely used by large beetles (Geotrupidae, namely *Geotrupes stercorarius* L., *Anoplotrupes stercorosus* Scriba, *Trypocopris alpinus* Sturm & Hagenbach), whereas control areas were virtually avoided (143 individuals trapped versus 7).

**Table 3:** Effects of pastoral practices on dung beetles.

			**MMS**			**TNCA**		
	**Dung beetle variables**	***Year***	**Est.**	**Wald**	***p***	**Est.**	**Wald**	***p***
Community indices	**N_DB_**	2011	-0.01	-0.03	N.S.	-0.53	-3.26	**<0.01**
	Distribution: NEG BINOMIAL	2012	0.34	2.64	**<0.01**	1.00	8.55	**<0.001**
	**S_DB_**	2011	-0.05	-0.58	N.S.	-0.13	-1.85	N.S.
	Distribution: POISSON	2012	0.23	2.97	**<0.01**	0.87	14.70	**<0.001**
	**H_DB_**	2011	-0.08	-1.57	N.S.	-0.06	-1.06	N.S.
	Distribution: GAMMA	2012	-0.22	3.77	**<0.001**	0.57	10.91	**<0.001**
Taxonomic diversity indices	**Δ**	2011	-0.23	-3.09	**<0.01**	-0.05	-0.50	N.S.
	Distribution: GAMMA	2012	0.15	2.20	**<0.05**	0.16	2.25	**<0.05**
	Δ*	2011	-0.60	-2.57	**<0.05**	-0.04	-1.85	**<0.05**
	Distribution: GAMMA	2012	0.04	1.11	N.S.	0.01	0.79	N.S.
	**Δ+**	2011	-0.03	-1.25	N.S.	-0.05	-2.30	**<0.05**
	Distribution: GAMMA	2012	-0.05	1.34	N.S.	0.06	3.38	**<0.001**
Functional diversity indices	**FD**	2011	-0.01	-3.09	N.S.	-0.15	-2.87	**<0.01**
	Distribution: GAMMA	2012	0.23	4.86	**<0.001**	0.72	19.90	**<0.001**

Treatment factor estimates and statistical significance (GLMM) for dung beetle community parameters (N_DB_, abundance; S_DB_, species richness; H_DB_, Shannon diversity index), taxonomical and functional diversity indices (Δ, taxonomic diversity; Δ*, taxonomic distinctness; Δ+, average taxonomic distinctness; FD, functional diversity) between treatment and control sites. Significant comparisons are in bold type. In this parameter estimation analysis, the control sites were used as the reference category.

**Figure 2:**
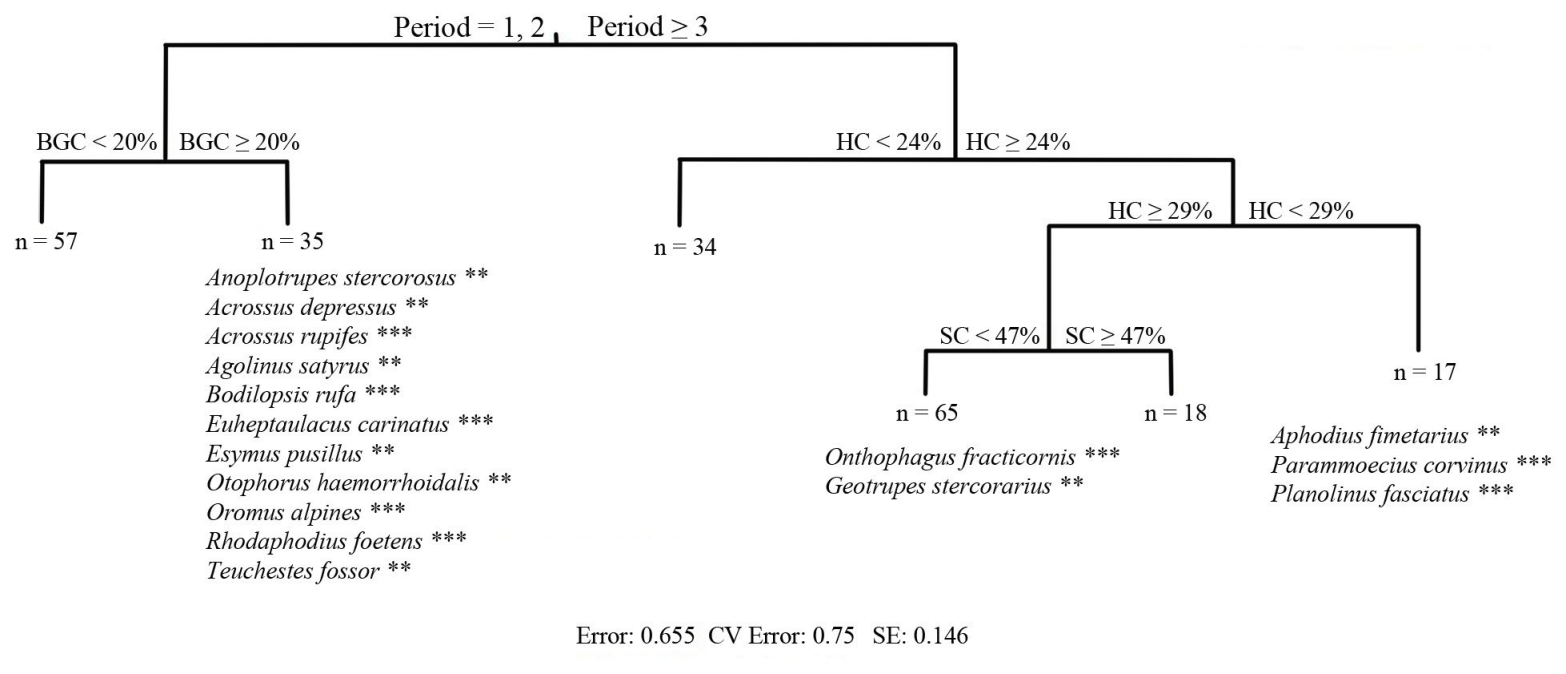
Multivariate Regression Trees and associated Indicator Value analyses. MRT for dung beetle abundance within TNCA one year after treatment. Numbers below each branch end give the number of traps. Only species which are significantly associated with one of the branches are shown. Statistical significance was obtained by Monte Carlo randomization tests (1000 runs). The model has an error term = 0.655 and crossvalidated error = 0.75 (0.146 SE). Period, *BGC, HC*, and *SC* represent the dung beetle sampling period, the percentage of bare ground cover, the herbaceous layer cover, and the shrub layer cover, respectively.

Five vegetation variables, notably percentage of shrub, herbaceous, and bare ground cover, average height of the herbaceous layer, and Shannon diversity index were significantly different between TNCA and control sites and were therefore used in MRT. The first dichotomy in MRT separated the first two sampling periods (early season periods) from the last three periods (late season periods) (Figure 2). This result reflected a variation in the seasonal abundance distribution of dung beetles, most of which were collected in September 2012. The branch of the first two sampling periods showed a successive node regarding different percentage of bare ground cover (i.e. with more or less than 20 %). The branch of the last three sampling periods showed three successive dichotomies regarding percentage of herbaceous and shrub cover. The IndVal procedure identified indicator species belonging to all three nesting behaviour categories: eleven early season species preferred sites with more bare ground cover gaps, three species preferred sites with herbaceous cover between 24 and 29 %, and two species selected sites having more than 29 % of herbaceous cover and less than 47 % of shrub cover.

Two vegetation variables, percentage of shrub cover and the percentage bare ground cover, were significantly different between MMS and control sites and were therefore used in MRT: in this case, however, no dichotomy based on vegetation variables was identified. 

## Discussion

In grassland restoration practices using livestock, complete vegetation restoration is usually achieved after some years [8]. However, this study showed that, by using appropriate pastoral practices, promising results may be obtained also in the short-term both on vegetation structure and, above all, on dung beetles. Furthermore, we were able to compare different pastoral practices (MMS and TNCA) in terms of the extent of changes to both dung beetles and vegetation, to compare the response of different ecosystem components (dung beetles or vegetation) to the treatments, and to assess the response of dung beetles according to different vegetation variables. In summary, TNCA were the most efficient pastoral practice, dung beetles responded most quickly to the treatments, and removal of shrubs allowed the quickest response of dung beetles.

### TNCA are more efficient than MMS

#### Effects on vegetation

Short-term effects produced by the use of MMS were the reduction of shrub cover and the increase of bare ground near supplement blocks. The average area modified by cattle (69 m^2^) was slightly wider than that measured by Probo et al. [38] (45m^2^) with free-ranging cattle, probably because MMS in this study were placed within a smaller area that made MMS more easily visible and usable by cattle. Vegetation *N* index and forage pastoral value did not show a significant increase one year after treatment, likely because re-colonization patterns and changes in vegetation composition are slow processes at high altitudes [20]. 

Short-term effects produced by TNCA were more intense than those produced by MMS. Indeed, the extent of modified areas was much wider in TNCA (each TNCA extended for about 1100 m^2^, on average) and this practice not only significantly reduced the cover of shrubs and increased the percentage of bare ground, but also increased the cover and the height of the herbaceous layer, Shannon diversity index and the pastoral value of the forage. The pronounced decrease in the shrub cover within TNCA made the proportion of different plant species more uniform within the plant community and therefore caused an increase in Shannon diversity index and in the forage pastoral value.

Differences between the two practices were probably related to the more intense effect of trampling, grazing, and fertilization produced within TNCA with respect to MMS sites. In particular, trampling caused serious and more widespread mechanical damage to the branches of shrubs within TNCA than at MMS sites, where the effect was concentrated only within a few meters around MMS blocks. Also, as observed in similar environments [8], both *J. nana* and *R. ferrugineum* did not show any sign of re-sprouting. The more intense deposition of faeces within TNCA caused a stronger effect of fertilization than at MMS sites, which could explain the more rapid increase in cover and height of the herbaceous layer one year after treatment. Nevertheless, this effect was not evident through a significant increase in the vegetation *N* index, probably because this indirect vegetation index is more sensitive to trophic changes in vegetation over longer periods [39]. 

#### Effects on dung beetles

Immediately after treatment, the disturbance effects produced by cattle on dung beetle assemblages at MMS sites were detectable, although they were rather limited. Indeed, only a moderate, though significant, reduction in two taxonomic diversity indices was detected at MMS with respect to control sites. The disturbance effects caused by cattle at TNCA were more intense, given that a significant decrease in abundance, functional diversity index, taxonomic distinctness, and average taxonomic distinctness was detected in 2011. Within TNCA, cattle were at high density, hence resembling a case of overgrazing. These results were therefore consistent with a previous study which demonstrated that cattle overgrazing is detrimental and may represent a threat to the conservation of alpine dung beetles [18]. On the whole, the instantaneous impact produced by treatments in 2011 exerted a negative, temporary effect on a few diversity parameters. In contrast, in 2012, one year after the treatments, the effects on dung beetles dramatically reversed, changing from slightly detrimental to beneficial. Indeed, both at MMS and TNCA sites, dung beetle abundance, species richness, functional diversity, and taxonomic diversity significantly increased with respect to control sites. The effect was particularly strong within TNCA, where Shannon diversity index and average taxonomic distinctness also significantly increased. This response may depend on the extent of the area modified by cattle, which was, on average, more than ten times larger in TNCA than in MMS sites. Indeed, Numa et al. [40] asserted that patch size, shape, and degree of connectivity with other patches are important factors in sustaining species-rich dung beetle populations.

### Dung beetles respond quicker than vegetation

Our results confirmed that re-colonization by herbaceous vegetation is a slow process in sub-alpine shrub-encroached grasslands. The main limiting factors are attributed to the limited seed bank of many grassland species under a dense shrub canopy [8] and to the rate-limitation of biochemical processes, growing season, and vegetation cycles due to low temperature at high altitudes [20]. Dung beetles responded quicker, with a significant increase in all biodiversity dimensions investigated with respect to control sites just one year after the treatment. In this context, the increase of large tunneller species (i.e. *G. stercorarius*, *A. stercorosus*, and *T. alpinus*) in TNCA during the second year may have increased seed dispersal as well as seed survival (and therefore plant recruitment) by reducing seed predation and mortality due to pathogens [15,16,41,42]. However, in addition to tunnellers, small and large dung and soil-ovipositing dwellers may also have an important functional role, and there is a complementarity among different functional groups in terms of ecological function [42]. An experimental manipulation of temperate dung beetle assemblages carried out by Rosenlew and Roslin [43] showed that dung decomposition is reduced by 12 % excluding small Aphodiidae dwellers. These beetles are dominant in temperate region [31,44], as confirmed in our study, and may contribute to ecosystem functioning, although often unevenly. Besides, the activity of Aphodiidae species at the dung-soil interface determines seed relocation in the top soil layers, which may support the germination conditions required by meso-eutrophic plants.

Some studies [15,45,46] indicated that the effect of biodiversity on ecosystem processes may be better explained by using different biodiversity dimensions such as functional diversity, taxonomic diversity, and different organizational levels of species diversity. The increase in diversity measures in our treatment areas might have beneficial consequences for the ecosystem given the key ecological role of dung beetles. In particular, the higher dung beetle abundance and species richness observed in MMS and TNCA with respect to control sites may indicate a “concave-up” relationship [41] with ecosystem functions such as dung removal, nutrient cycling, soil aeration, secondary seed burial, plant regeneration, and N and P plant uptake [13–16,42,47]. Consequently, we suggest that the effects of these pastoral practices may support the medium-term restoration of herbaceous cover with positive effects on dung beetle communities which prefer open habitats. The resulting increase in dung beetle abundance and diversity may have a positive effect on meso-eutrophic grassland restoration. 

### Shrub removal enables dung beetle colonization

Multivariate Regression Trees and Indicator Species Analysis showed that one year after the TNCA treatment, most dung beetle species preferred bare ground sites, meaning that shrub removal is the first structural vegetation change they require for colonizing the new open habitat. MRT carried out on 2012 data successfully identified dichotomies based on vegetation variables for TNCA, but not so for MMS, confirming the idea that structural vegetation changes induced by TNCA were stronger. MRT and associated IndVal analyses confirmed that changes in TNCA dung beetle assemblages were mainly modulated by seasonality, but also showed that of a total of 21 species sampled, eleven preferred areas which had been deprived of shrub vegetation and were characterized by more than 20 % of bare ground. Our results show therefore that dung beetles prefer habitat from which shrubs have been partly removed by pastoral practices. These results are in keeping with those of Tocco et al. [48], who demonstrated that shrub habitat was suboptimal for dung beetles. The removal of shrubs seems to be particularly appreciated by large beetles like *G. stercorarius*, known to be threatened by land use changes [43], which were collected in large quantities in TNCA. This in turn is likely to represent a positive effect on ecosystem functions, given that high species richness and abundance of large beetles seem to exert the major effect on key ecological processes [42]. The use of pastoral practices may also have positive conservation effects because most species in the Alps are associated with open habitats [44,48,49]. 

These short-term (one year) results on the effects of pastoral practices to reverse shrub-encroachment and restore sub-alpine open habitats are promising. With respect to control sites, both practices significantly changed the structure of vegetation by removing shrubs and increasing open areas. Both practices, after having caused a moderate decrease in dung beetle diversity immediately after the treatment, caused a significant increase one year later. In particular, TNCA appeared to supply better results than MMS, both in terms of spatial extent and efficacy of the restoration of plant and dung beetle communities. Furthermore, TNCA led to an optimal vegetation structure for the dung beetle community supporting redundancy of functional group species, as confirmed by the increase of the functional diversity index, with possible implications in maintaining and improving ecosystem functions. These pastoral practices for the restoration of open habitats appeared to be less invasive than those carried out in other research, such as prescribed burning [50], which produced negative effects on fauna immediately after their implementation, or mowing, which may be more costly than grazing and unfeasible by machinery in rugged alpine areas [51]. Both practices may be a viable management option to be considered for the restoration of sub-alpine open habitats within agri-environmental schemes. Indeed, even though the strategic placement of MMS appeared to be less promising in terms of the results produced than TNCA, it is more sustainable as it requires less labor. Furthermore, after the implementation of these practices, areas should be grazed regularly in order to maintain and improve the open habitats restored in the long-term. In summary, we believe these results are noteworthy because it is the first time that these practices for the restoration of open habitats have been tested in a framework of ecosystem functionality, taking into account the effect produced by cattle both on vegetation and dung beetles, which are one of the main mediators of dung nutrient redistribution in soil and are therefore responsible for the ecosystem function of these semi-natural grasslands. 

## Supporting Information

Appendix S1 **Details of the vegetation and dung beetle variables used in GLMM analyses.** Table S1, Classification of the species sampled according to four functional diversity traits. The nesting behaviour (N1, dung-ovipositing dweller; N2, soil-ovipositing dweller; N3, tunneller), weight (W1, < 0.005 g; W2, 0.005 g ≤ x ≤ 0.01 g; W3, 0.01 g ≤ x ≤ 0.5 g; W4, > 0.5 g), external protoracical leg length (LL1, < 0.38 mm; LL2, 0.38 mm ≤ x ≤ 0.41 mm; LL3, > 0.41 mm) and tibia width across apex of third tooth and external side (TW1, < 0.125 mm; TW2, 0.125 mm ≤ x ≤0.135 mm; TW3 > 0.135 mm).
(DOC)
