# Supplementary material for: Pastoral Practices to Reverse Shrub Encroachment of Sub-Alpine Grasslands: Dung Beetles (Coleoptera, Scarabaeoidea) Respond More Quickly Than Vegetation
Source: PLoS One. 2013 Dec 16;8(12):e83344. doi: 10.1371/journal.pone.0083344 (PMC3865246; doi:10.1371/journal.pone.0083344)
Supplement: Appendix S1 — Details of the vegetation and dung beetle variables used in GLMM analyses. Table S1, Classification of the species sampled according to four functional diversity traits. The nesting behaviour (N1, dung-ovipositing dweller; N2, soil-ovipositing dweller; N3, tunneller), weight (W1, < 0.005 g; W2, 0.005 g ≤ x ≤ 0.01 g; W3, 0.01 g ≤ x ≤ 0.5 g; W4, > 0.5 g), external protoracical leg length (LL1, < 0.38 mm; LL2, 0.38 mm ≤ x ≤ 0.41 mm; LL3, > 0.41 mm) and tibia width across apex of third tooth and external side (TW1, < 0.125 mm; TW2, 0.125 mm ≤ x ≤0.135 mm; TW3 > 0.135 mm). (DOC) [file pone.0083344.s001.doc]

**Appendix S1. Details of the vegetation and dung beetle variables used in GLMM analyses.**

VEGETATION VARIABLES

Average vegetation *N* index (*N_average_*)

An indirect vegetation index was calculated to evaluate the overall effect of fertilization produced by the cattle, as the exact amount of dung and urine excreted in the surrounding areas of MMS and within the TNCA was extremely difficult to assess. *N_average_*, weighted for species relative abundance (SRAi), was computed for each transect according to the equation:

where *N_i_* is the *N* nutrient value for the species *i*.

Forage pastoral value (PV)

Each species was also classified according to the Index of Specific Quality (ISQ) [3]. The ISQ is based on preference, morphology, structure, and productivity of the plant species found in the Western Italian Alps, and it ranges from 0 to 5. In each transect, forage pastoral value (PV), a synthetic value summarizing forage yield and nutritive value ranging from 0 to 100 was calculated [3]. Forage pastoral value is weighted for species relative abundance and is calculated using the following equation:

where ISQ*_i_* is the ISQ value for the species *i*.

DUNG BEETLE VARIABLES

Taxonomic diversity indices

*Taxonomic diversity (Δ)* is the average taxonomic distance between any two organisms, chosen at random from the sample [27].

$$\Delta=\left[\sum\sum_{i<j}\omega_{ij}x_{i}x_{j}\right]/\left[n(n-1)/2\right]$$

where ω are taxonomic distances among taxa i and j, x are species abundance, and n is the total number of individuals in the sample

*Taxonomic distinctness (Δ*)* is defined as a measure of pure taxonomic relatedness, being the average phylogenetic path length between any two randomly chosen individuals within a sample [27].

$$\Delta*=\left[\sum\sum_{i<j}\omega_{ij}x_{i}x_{j}\right]/\left[\sum\sum_{i<j}x_{i}x_{j}\right]$$

where ω are taxonomic distances among taxa *i* and *j*, and x are species abundances.

*Average taxonomic distinctness (Δ+)* represents the case of taxonomic distinctness (Δ*) when calculated using presence/absence data and is based on the taxonomic distance through a classification tree between every pair of species within a sample [28].

$$\Delta^{+}=\left[\sum\sum_{i<j}\omega_{ij}\right]/\left[s(s-1)/2\right]$$

where ω are taxonomic distances among taxa *i* and *j* and *s* is the number of species present.

Functional diversity index (FD)

The functional traits used to classify the sampled species in order to calculate the FD were: nesting behaviour (according to [13,30,31]), weight, and two protoracical leg allometries. These traits were given by the ratio between the body size (maximum width of pronotum) and two different protoracical leg allometries (external protoracical leg length and tibia width across the apex of the third tooth and external side), which were not correlated (correlation was assessed with Pearson’s correlation test). Weight, body size and linear measurements of the prothoracical tibia for ten specimens within each species collected were recorded. We acquired images of tibia and pronotum by the modular zoom system Leica Z16Apo macroscope connected to a Leica DFC320 digital camera (Leica Microsystems AG, Wetzlar, Germany). Measurements were then taken using the LAS (Leica® Application Suite) software v 2.5.0.

**Table S1.** **Classification of the species sampled according to four functional diversity traits**. The nesting behaviour (N1, dung-ovipositing dweller; N2, soil-ovipositing dweller; N3, tunneller), weight (W1, < 0.005 g; W2, 0.005 g ≤ x ≤ 0.01 g; W3, 0.01 g ≤ x ≤ 0.5 g; W4, > 0.5 g), external protoracical leg length (LL1, < 0.38 mm; LL2, 0.38 mm ≤ x ≤ 0.41 mm; LL3, > 0.41 mm) and tibia width across apex of third tooth and external side (TW1, < 0.125 mm; TW2, 0.125 mm ≤ x ≤0.135 mm; TW3 > 0.135 mm).

| **Dung beetle species** | **W1** | **W2** | **W3** | **W4** | **N1** | **N2** | **N3** | **LL1** | **LL2** | **LL3** | **TW1** | **TW2** | **TW3** |
| --- | --- | --- | --- | --- | --- | --- | --- | --- | --- | --- | --- | --- | --- |
| *Acrossus depressus*(Kugelann, 1792) |  | x |  |  |  | x |  |  |  | x | x |  |  |
| *Acrossus rufipes*(Linnaeus, 1758) |  |  | x |  |  | x |  |  |  | x |  |  | x |
| *Agolinus satyrus* (Reitter, 1892) |  | x |  |  | x |  |  |  | x |  |  | x |  |
| *Amidorus spp.* (Mulsant & Rey, 1870) |  |  | x |  |  | x |  |  |  | x |  | x |  |
| *Anoplotrupes stercorosus* (Scriba, 1796) |  |  |  | x |  |  | x | x |  |  |  |  | x |
| *Aphodius fimetarius* (Linnaeus, 1758) |  | x |  |  |  | x |  |  |  | x | x |  |  |
| *Bodilopsis rufa* (Moll, 1782) |  |  | x |  | x |  |  |  | x |  | x |  |  |
| *Colobopterus erraticus*(Linnaeus, 1758) |  | x |  |  |  |  | x |  |  | x |  | x |  |
| *Coprimorphus scrutator* (Herbst, 1789) |  |  |  | x |  |  | x | x |  |  |  | x |  |
| *Esymus pusillus* (Herbst, 1789) | x |  |  |  | x |  |  |  |  | x | x |  |  |
| *Euheptaulacus carinatus* (Germar, 1824) | x |  |  |  | x |  |  | x |  |  |  | x |  |
| *Geotrupes stercorarius* (Linnaeus, 1768) |  |  |  | x |  |  | x | x |  |  |  |  | x |
| *Onthophagus baraudi* (Nicolas, 1964) |  |  | x |  |  |  | x |  | x |  |  | x |  |
| *Onthophagus fracticornis* (Preyssler, 1790) |  |  | x |  |  |  | x |  |  | x |  |  | x |
| *Oromus alpinus* (Scopoli, 1763) |  | x |  |  |  | x |  |  | x |  |  | x |  |
| *Otophorus haemorrhoidalis* (Linnaeus, 1758) |  | x |  |  | x |  |  |  | x |  | x |  |  |
| *Parammoecius corvinus* (Erichson, 1848) | x |  |  |  | x |  |  |  |  | x | x |  |  |
| *Planolinus fasciatus* (Olivier, 1789) | x |  |  |  | x |  |  |  | x |  |  | x |  |
| *Rhodaphodius foetens* (Fabricius, 1787) |  |  | x |  | x |  |  |  |  | x | x |  |  |
| *Teuchestes fossor* (Linnaeus, 1758) |  |  | x |  |  | x |  | x |  |  |  | x |  |
| *Trypocopris alpinus* (Sturm & Hagenbach, 1825) |  |  |  | x |  |  | x | x |  |  |  |  | x |
